# Impacting Microbial Communities and Absorbing Pollutants by *Canna Indica* and *Cyperus Alternifolius* in a Full-Scale Constructed Wetland System

**DOI:** 10.3390/ijerph16050802

**Published:** 2019-03-05

**Authors:** Yinghai Wu, Tao He, Chen Chen, Xiaohang Fang, Dongyang Wei, Jing Yang, Renduo Zhang, Rui Han

**Affiliations:** 1 College of Marine and Civil Engineering, Dalian Ocean University, Dalian 116023, China; wuyinghai@dlou.edu.cn; 2 Guangdong Provincial Key Laboratory of Environmental Pollution Control and Remediation Technology, Sun Yat-sen University, Guangzhou 510275, China; 3 South China Institute of Environmental Science, Ministry of Environment Protection, Guangzhou 510655, China; hetao@scies.org (T.H); chenchen7@scies.org (C.C.); fangxiaohang@scies.org (X.F.); weidongyang@scies.org (D.W.); yangjing@scies.org (J.Y.); 4 College of Marine Technology and Environment, Dalian Ocean University, Dalian 116023, China

**Keywords:** wetland plant, phytoremediation, microbial distribution, functional gene, biological inhibition, high-throughput

## Abstract

Wetland plants that cover the wetlands play an important role in reducing pollutants. The aim of this study was to investigate the effect of two plant species on microbial communities and nitrogen-removal genes and to evaluate the contributions of absorbing pollutants by *Canna indica* (CI) and *Cyperus alternifolius* (CA) to the removal performance in both a vertical subsurface flow constructed wetland and a horizontal subsurface flow constructed wetland, which were part of a full-scale hybrid constructed wetland system. The microbial assemblages were determined using 16S rRNA high-throughput sequencing. Results showed that the presence of CI and CA positively affected microbial abundance and community in general and which was positive for the total bacteria and ammonia nitrogen removal in the CWs. The higher abundance of Nitrospirae appeared in the non-rhizosphere sediment (NRS) than that in the rhizosphere sediment (RS). More denitrification genes were found in NRS than in RS. The copy numbers of *narG*, *nirS* and *nosZ* genes for CA were higher than those for CI. Wetland plant species can significantly (*P* < 0.05) affect the distribution of microbial communities in RS. Plant selection is important to promote the development of microbial communities with a more active and diverse catabolic capability and the contribution of plant absorption to the overall removal rate of wetland system can be neglected.

## 1. Introduction

Constructed wetlands (CWs) have be reported as energy saving, reliable operation and convenient for maintenance in many studies [1,2]. The practical application of the system has been developed from single types to hybrid systems recently. A hybrid constructed wetland (HCW) is composed of two or more types of CWs to take the advantages of individual CWs and complement each other [1].

Wetland plants, as an important component of constructed wetlands, have attracted worldwide attention [2]. The root system of wetland plants can promote contaminant binding, more carbon sources and more active redox environment [3]. In turn, it directly or indirectly stimulates soil microbial activity. The roles of wetland plants that covered CWs were thought to be well established [4]. Nevertheless, many wetland plant species usually exhibit different characteristics, such as biomass, root densities and root exudates [3,5]. Previous studies demonstrated that plant offered more suitable conditions via root and root exudates (e.g., the radial oxygen leakage (ROL) and excretion of carbon) for bacterial growth and thereby increased the bacterial population [3,6]. In contrast, plants root and exudates could also play a role in reducing nitrification by an inhibitory root exuded compound [7] or reducing denitrification by increasing oxygen which was less favorable for denitrifiers in wetlands. Therefore, the practical plantation is still premature as we lack the appropriate knowledge of microbial community shift with RS and NRS and species [4,8].

At present, many studies on wetlands are based on natural wetlands and laboratory scale [2,4,9]. Few studies had been carried out on plants in practical full-scale engineering systems. Most of previous studies investigated the single removal of nutrients or heavy metals by plants [2,10]. The relations between plant biomass and pollutants concentration in the tissues and their impact factors had not showed consistent results [2,11,12]. Moreover, the allocation for the metals removal in different plant tissues had not been clearly identified.

The objectives of the present study were: (i) to investigate effect of two plant species on microbial community, nitrifying, denitrifying and anaerobic ammonia oxidizing (anammox) bacteria in both a vertical subsurface flow constructed wetland (VSFCW) and a horizontal subsurface flow constructed wetland (HSFCW), which were part of a full-scale hybrid constructed wetland (HCW) system; (ii) to evaluate the contributions of absorbing nutrients and heavy metals by *Canna indica* (CI) and *Cyperus alternifolius* (CA) to the removal performance in both a VSFCW and a HSFCW.

## 2. Materials and Methods

### 2.1. System Description and Sampling Procedure

**Figure 1:**
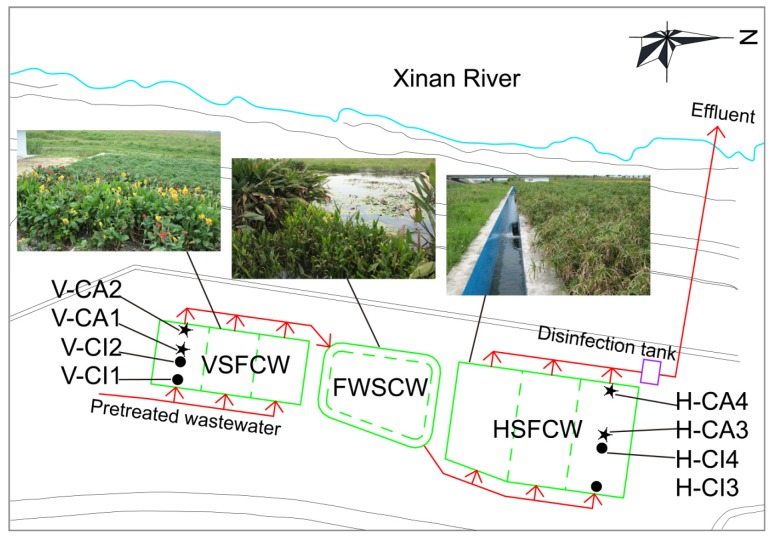
Geographic location, layout, photos and sampling locations of the study hybrid constructed wetland (HCW) system. V = VSFCW = vertical subsurface flow constructed wetland; FWSCW = free water surface constructed wetland; H = HSFCW = horizontal subsurface flow constructed wetland. Arrows show water flow direction in the system. Each circle represents the sampling locations of *Canna indica* (CI). Each star represents the sampling locations of *Cyperus alternifolius* (CA).

The HCW system was constructed in the northwest of Foshan City, Guangdong Province in Southern China (112°58′16.74″ E, 23°12′37.70″ N) and had operated for six years and two months before sampling. Briefly, the HCW system was composed of a VSFCW, a free water surface constructed wetland (FWSCW) and a HSFCW in series (Figure 1). A detail of the HCW design can be seen in Supplementary Material (SM).

The vegetation samples were collected from the VSFCW and HSFCW in June 6, which approached the annual harvesting date (Figure 1). Four CI and four CA were collected which heights represented the average growth of whole plants in the zones. CI samples were numbered V-CI1, V-CI2, H-CI3 and H-CI4, CA samples were numbered V-CA1, V-CA2, H-CA3 and H-CA4 (Figure 1). The water samples were sampled at inlet and outlet of CWs. Both rhizosphere sediment (RS) samples and non-rhizosphere sediment (NRS) samples were collected at depth of 20 cm, about 0.5m horizontal distance between them. A detail of the sampling procedure can be seen in Supplementary Material (SM).

### 2.2. DNA Extraction, Quantitative Real-Time Polymerase Chain Reaction and High-Throughput Sequencing

The extraction, detection and determination procedures of Genomic DNAs of the sediment samples can be seen in Supplementary Information.

The copy numbers of nitrogen transformation genes were determined by the quantitative real-time polymerase chain reaction (PCR) method. For quantification of 16S rRNAs and functional genes, which include total bacteria 16S rRNAs, hydrazine synthase (*hzsA*), AOB ammonia monooxygenase (*amoA*), membrane-bound nitrate reductase (*narG*), dissimilatory cd1-containing nitrite reductase (*nirS*) and nitrous oxide reductase (*nosZ*) genes, MyiQ5 (BIO-RAD, Hercules, CA, USA) based on SYBR Green II method was used. The primer pairs and amplification programs are summarized in the Table S1 (in Supplementary Information). The details of PCR mixture and PCR procedure can be seen in Supplementary Information.

Primers for sequencing were 515F (5′-GTG CCA GCM GCC GCG GTA A-3′) and 806R (5′-GGA CTA CHV GGG TWT CTA AT-3′), with different barcodes for the V4 region of bacterial and archaeal 16S rRNA gene [13]. The detail of PCR mixture and PCR procedure can be seen in Supplementary Information. A mixture of the amplicons was then used for sequencing on the Illumina MiSeq platform (paired-end 250-bp mode) at the Guangzhou Magigen Biotechnology Co. Ltd. The sequences have been deposited in the NCBI Sequence Read Archive under the accession number SRP186692.

### 2.3. High-Throughput Sequencing Data Analysis

Reads with a quality less than 30 at the 3’ end were trimmed. The quality sequences were clustered into operational taxonomic units (OTUs) at the 97% similarity level. Taxonomic assignment was determined at the 80% threshold. The detail of high-throughput sequencing data analysis can be seen in Supplementary Information.

Relative abundance (%) of individual taxa within each community was estimated by comparing the number of sequences assigned to a specific taxon versus the number of total sequences obtained for a sample. Calculations of alpha-diversity (including Faith’s phylogenetic diversity (PD), Chao1, observed species, Shannon and Simpson) and beta-diversity (Bray−Curtis distance) metrics were based on a subset of 10,335 randomly selected sequences from each sample. Bray−Curtis-based principal coordinate analysis (PCoA) was used to show the differences among the sediment samples of the two types of CWs. Beta diversity was also evaluated with the bacterial community data to examine the differences of community patterns related to CW structures and sampling locations via the Venn diagram.

### 2.4. Plant Biomass

All the vegetation samples were transported to the laboratory in cold, washed and separated to roots, rhizomes, stem, leaves and flower. To measure dry weight, vegetation samples were dried at 75 °C for 48 h to a constant weight [14,15]. The dry weights were determined, and the dried samples were powered and stored for further analyses.

### 2.5. Physicochemical Analysis

The water samples were used to measure pH, dissolved oxygen (DO), redox potential (*E_h_*), chemical oxygen demand (COD), ammonium nitrogen (NH_4_-N), nitrate nitrogen (NO_3_-N), total nitrogen (TN), total phosphorus (TP), Cd, Cu, Ni and Zn. The sediment samples were used to measure pH, *E_h_*, total organic carbon (TOC), NH_4_-N, NO_3_-N, TN, TP, Cd, Cu, Ni and Zn. The plant samples were used to measure TN, TP, Cd, Cu, Ni and Zn. The measurements were repeated for three times for each sample. The methods used to measure the physicochemical indices of the samples were described in Supplementary Information.

### 2.6. Statistical Analyses

All statistical analyses were implemented using SPSS 18.0 software (SPSS, Inc., Chicago, IL, USA). Kolmogorov-Smirnov test was used to determine whether the data distribution is normal. An independent sample t-test was used to examine the difference between the biomass of same plant species in different types of CWs. Pearson correlation coefficients were calculated to evaluate the relationships between biomass and physicochemical parameters. One-way analysis of variance (ANOVA) was performed to compare differences in physicochemical properties of water among the sampling sites, as well as physicochemical properties of sediment, nutrients concentration, heavy metal concentration and functional genes numbers. Post-hoc tests with Duncan’s statistics at *p* = 0.05 was performed to analyze the differences between groups of data.

## 3. Results

### 3.1. Sediment Microbial Diversity and Community Composition

High-quality sequences of 397,181 were acquired from the 24 sediment samples, with a range of 10,335 to 24,652 sequences per community (Table S2 in Supplementary Information). In total, 154,009 OTUs were identified, of which 12.5% were detected in only a single sample. Rarefaction analysis indicated that the full extent of microbial diversity was generally high in the sediment samples of the CWs (Figure S1 in Supplementary Information).

**Figure 2:**
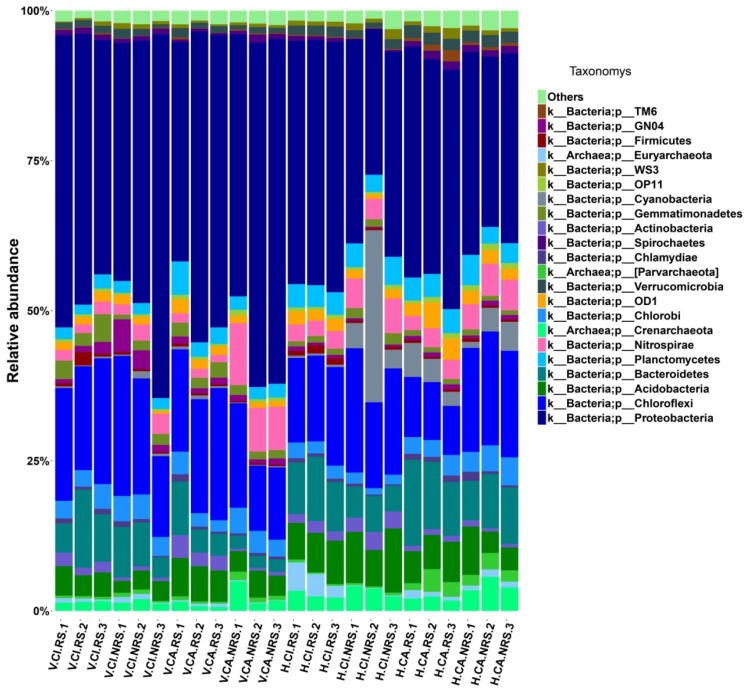
Relative abundance (%) of dominant microbial taxa across all analyzed sediments revealed by 16S rRNA MiSeq sequencing, at phylum level, mean relative abundance > 1%. In the sample number: V = VSFCW; H = HSFCW; CI = *Canna indica*; CA = *Cyperus alternifolius*; RS = rhizosphere; NRS = non-rhizosphere; three duplicates in each zone were numbered with 1, 2 and 3.

In sediment communities, *Bacteria* was dominated (>94.3%) with a low proportion of *Archaea* (<5.7%) in the VSFCW and HSFCW. The Relative abundance of *Archaea* (4.7%) in CA groups was higher than that (4.7%) in CI groups (Figure 2). Among the 22 bacterial phyla identified, *Proteobacteria* were predominant (24.4–60.6%) across all the sediments, with other abundant phyla, such as *Chloroflexi* (8.2–23.3%), *Acidobacteria* (3.1–10.6%), *Bacteroidetes* (2.0–14.3%) (Figure 2). Remarkably, the majority of archaeal sequences were affiliated with *Crenarchaeota*, which abundance reached the maximum in the NRS of CA in VSFCW. The abundance of *Nitrospirae* obviously fluctuated among all samples. The abundance of *Nitrospirae* in the NRS was higher than that in the RS and its abundance in CA was higher than that in CI. The abundance of *Cyanobacteria* showed higher in the HSFCW than the VSFCW.

**Figure 3:**
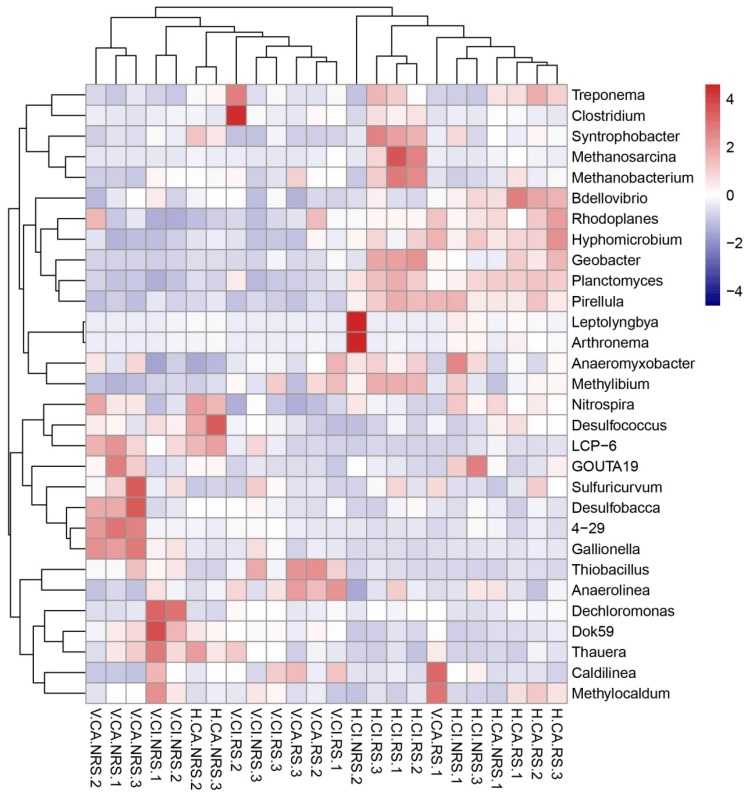
Relative abundance (%) of top 30 microbial genera across the two wetlands revealed by 16S rRNA MiSeq sequencing. The samples numbers were shown in Figure 2.

At the genus level, members from *Nitrospira*, *Leptolyngbya*, *Thiobacillus* and *Geobacter* were most frequently detected (Figure 3). *Leptolyngbya* showed its highest abundance (17 ± 3.0%) in NRS of CI in VSFCW and the abundance in NRS was higher than that in RS of two plant species. *Thiobacillus* showed the highest abundance (19.8 ± 1.7%) in CA RS in VSFCW and the abundance in RS was higher than that in NRS for two plant species. The relative abundance of *Nitrospira* in NRS was higher than that in RS. *Geobacter* showed the opposite trend with *Nitrospira* in NRS and RS in two plants, the abundance in RS is higher than that in NRS in two plants. *Dechloromonas* showed higher abundance in V.CI.NRS samples.

The richness and diversity indexes No. of OTUs, Faith’s PD and chao 1 ranged from 4109 to 5145, from 269 to 351 and from 11,533 to 16,279, respectively (Table S2 in Supplementary Information). For CI in the HSFCW and CA in VSFCW, the microbial diversity was significantly higher in the RS than that in the NRS (*p* < 0.005), for CA in the HSFCW and CI in VSFCW, the microbial diversity showed no significant difference in the RS and NRS (Table S2 in Supplementary Information).

**Figure 4:**
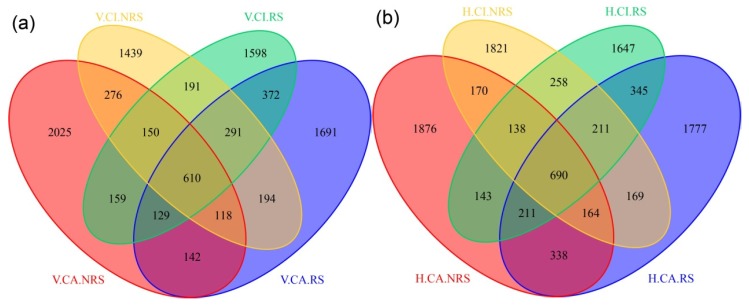
Venn diagram showing the unique and shared OTUs (0.03 phylogenetic distance). Venn diagram for four sampling zones in VSFCW (**a**) and four sampling zones HSFCW (**b**). The calculation of the Venn diagram was based on results of three duplicates in each zone. The samples numbers were shown in Figure 2.

Results of the Unifrac-based PcoA showed that the samples distributed in different parts of the data space (Figure S2 in Supplementary Information), indicating significant differences in sediment community compositions among the sampling sites. For VSFCW, the bacterial communities in the CI.NRS and the CA.NRS shared 1154 OTUs (20.2%) and 1402 OTUs (24.8%) were shared by two communities in the CI.RS and the CA.RS, respectively (Figure 4). For HSFCW, the bacterial communities in the CI.NRS and CA.NRS shared 1162 OTUs (18.8%) and 1457 OTUs (23.9%) were shared by two communities in the CI.RS and CA.RS, respectively. For two CWs, the OTUs was significantly higher in CA group than that in CI group. The results showed that the microbial diversity of CA group was higher than that of CI group.

### 3.2. Quantification of Microbial 16S rRNAs and Functional Genes

**Figure 5:**
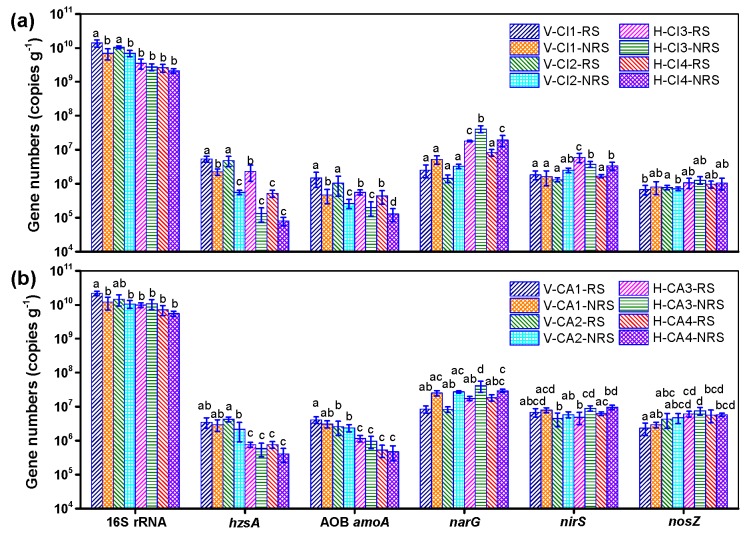
Quantitative analysis of microbial 16S rRNA and functional genes in the RS and NRS in VSFCW and HSFCW. (**a**) *Canna indica* (CI); (**b**) *Cyperus alternifolius* (CA). Error bars represent standard deviation calculated from three independent experiments. The samples numbers were shown in Figure 2.

Results of total bacteria 16S rRNAs, *hzsA*, AOB *amoA*, *narG*, *nirS* and *nosZ* genes of 16 sediment samples in the two wetlands are shown in Figure 5. For each plant, the abundance of these genes varied significantly among the eight sites (*p* < 0.05), except for *nirS* in the samples of CI. For CI, the copy numbers of total bacterial 16S rRNAs varied among 1.76 × 10^9^~1.70 × 10^10^ copies g^−1^ and showed significantly higher number in the RS than the NRS in VSFCW (Figure 5a). Similar differences were also presented for *hzsA* and AOB *amoA*. The copy numbers of *narG* and *nosZ* showed lower in the RS than the NRS although no significant differences were found in VSFCW and HSFCW. The copy numbers of *nirS* showed no significant differences between the RS and the NRS in VSFCW, showed irregular in HSFCW. For CA, the copy numbers of the total bacterial 16S rRNAs varied among 4.68 × 10^9^~2.51 × 10^10^, showed higher numbers in the RS than the NRS (Figure 5b). Almost similar differences were also presented for *hzsA* and AOB *amoA*. The copy numbers of *narG* as well as *nirS* were lower in the RS than the NRS, also significant differences only within HSFCW. The copy numbers of *nosZ* showed slightly lower in the RS than the NRS. The copy numbers of *narG*, *nirS* and *nosZ* genes for CA were higher than those for CI.

### 3.3. Physicochemical Properties

Physicochemical properties of wastewater and sediment are shown in Tables S3 and S4 (in Supplementary Information). The concentrations of all pollutant indicators decreased from the inlet to the outlet in each CW except for NO_3_-N. DO, *E_h_*, COD, NH_4_-N, TN and SO_4_^2−^ at the inlets of VSFCW were significantly different from those of HSFCW (*p* < 0.05), except pH, NO_3_-N and TP (*p* > 0.05). At the outlets, DO, *E_h_*, COD, NH_4_-N, NO_3_-N and TN were significantly different among the two types of CWs (*p* < 0.05), while pH, TP and SO_4_^2−^ were not significantly different (*p* > 0.05). At an average sewage flow of 1120 m^3^ during sampling period, the removal loads of COD, NH_4_-N, TN and TP in VSFCW were 37.6 g m^−2^ d^−1^, 4.22 g m^−2^ d^−1^, 3.59 g m^−2^ d^−1^ and 0.37 g m^−2^ d^−1^, respectively. While those in HSFCW were 3.69 g m^−2^ d^−1^, 0.78 g m^−2^ d^−1^, 3.28 g m^−2^ d^−1^, 0.12 g m^−2^ d^−1^, respectively.

### 3.4. Plant Biomass

The biomass (g DW m^−2^) of two wetland plants grown at the end of 150 days in the VSFCW and HSFCW of the HCW system was shown in Table 1. The biomass of roots, stems and leaves of CI was significantly higher than that of rhizomes and flowers (*p* < 0.05). The biomass of CI was higher than that of the CA in the VSFCW but did not reach a significant level (*p* > 0.05). The biomass of CI was significantly higher than that of CA in HSFCW (*p* < 0.05). The aboveground biomass of CI was significantly higher than that of the underground (*p* < 0.05) and the aboveground biomass of CA was lower than the underground biomass but was not significantly different (*p* > 0.05).

### 3.5. Allocation of Nutrient and Heavy Metal in Plant Tissues

**Figure 6:**
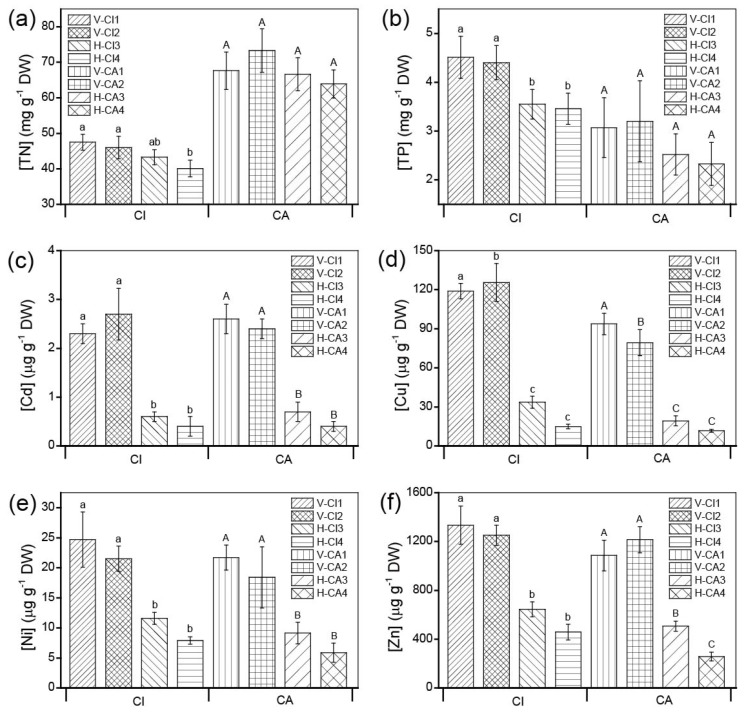
Nutrients concentration (mg g^−1^ DW), heavy metal concentration (μg g^−1^ DW) in roots of the two plants at the eight sites in the HCW system. (**a**) total nitrogen (TN); (**b**) total phosphorus (TP); (**c**) Cd; (**d**) Cu; (**e**) Ni; (**f**) Zn. The samples numbers were shown in Figure 1.

The concentrations of nutrients as well as heavy metals in CI roots were significantly different among four sites (*p* < 0.05 or *p* < 0.001) (Figure 6). Contrary to TP, TN concentrations in the roots of CI were lower than those in the roots of CA. The metals concentrations in the roots of CI were higher than those in roots of CA, except that Cd was almost similar between them. The concentrations of nutrients and heavy metals in rhizomes, leaves and flowers were shown in Figure S3 and Table S5. The concentrations of TN in other plant tissues of two plant species showed similar differences like those in roots. In brief, the TN concentrations in CI were lower than those in CA, whereas the concentrations of heavy metals in CI were higher than those in CA. The concentrations of nutrients were not significantly different between the underground and aboveground tissues of CI, as well as CA (*p* > 0.05), while four kinds of heavy metals in the underground tissues were significantly higher than the aboveground tissues.

## 4. Discussion

### 4.1. Microbial Community Shift With Different Samples

Proteobacteria as frequently the dominant phylum in all kinds of wetland, including the genera *Zoogloea*, *Comamonas*, *Thiobacillus*, *Nitrosospira*, *Denitratisoma*, *Azonexus* and *Azospira*, is usually considered played a vital role in the removal of organic matter and nitrogen [1]. More DO concentration (Table S3) in the influent leaded to more microbial abundances of *Proteobacteria* and *Cleroflexi* in VSFCW than in HSFCW (Figure 2), which contributed to the removal of a large number of organic and nitrogen pollutants in VSFCW. The copy numbers of total bacterial 16S rRNAs, *hzsA* and AOB *amoA* also showed similar patterns (Figure 5). Rich diversity of the microbial community and the high proportion of nitrogen-cycle bacteria were essential for nitrogen removal in this treatment system [16]. The higher relative abundance of *Chloroflexi* in all sites indicated the rapid growth of filamentous bacteria and their potential role in carbon cycling during litter decomposition and provided available carbon source to the heterotrophic bacteria [17]. The higher abundance of *Nitrospirae* which performed nitrite oxidation in the second step of nitrification in the NRS than that in the RS may be due to the inhibition of nitrifying bacteria by root exudates [7,8,18]. In addition, as plants need less energy to absorb ammonia nitrogen than nitrate nitrogen [19], relatively low concentration of ammonia nitrogen and relatively high concentration of nitrate nitrogen in rhizosphere may inhibit the metabolism of nitrifying bacteria. The higher abundance of *Nitrospirae* in CA than that in CI showed that CA has better nitrification ability. The microbial genera, such as *Geobacter*, *Methanosarcina*, *Nitrospira* and *Thauer* showed obviously different relative abundances between VSFCW and HSFCW in this study (Figure 3). Due to the different flow direction and oxygen carrying capacity of the influent for two CWs, the phenotype of microbial community was affected by different redox potentials in substrates, more aerobic bacteria, such as *Nitrospira* were found in VSFCW, while more anaerobic bacteria, such as *Methanosarcina* were found in HSFCW. More information about how wetland structure affected microbial phenotypes in the HCW systems studied can be seen in our previous studies [1].

Wetland plants could provide a large surface area for attached microbial growth and supply reduced carbon and oxygen in the rhizosphere [1]. Most previous studies reported plant presence positively affected microbial abundance and community [20,21]. Plant productivity and below ground biomass impacted anaerobic microbial metabolism in a potted plant study [9]. Nevertheless, the root exudates may have a direct impact on nitrogen cycling, as they may inhibit nitrification process by soil nitrifying microorganisms (Figure 2 and Figure 3) [8]. The root exudates of CA group had more strong inhibition compare to CI group. The denitrifying bacteria, *Dechloromonas* [22], showed higher abundance in V.CI.NRS samples (Figure 3), which indicates that some denitrifying bacteria may be inhibited by the ROL of root system. A few studies have found no significant difference in microbial communities between root and non-root systems [23,24].

Wetland plant species may significantly affect the distribution of microbial communities in RS. The CW systems planted with *Vetiveria zizanioides* or *Juncus effusus* L. showed much higher bacterial abundance but lower archaeal abundance [25]. There were significantly more bacteria on *P. australis* roots when compared to the roots of *Phalaries arundinacea* [26]. Denitrification strongly depends on both the presence of emergent plants and the denitrifier communities selected by different plant species [27]. Nevertheless, other studies showed some plants appear to exert little effect on the structure of microbial communities in constructed wetlands [28,29,30]. Those results indicates that plant selection is important to promote the development of microbial communities with a more active and diverse catabolic capability [31]. Higher microbial richness and diversity, as well as a higher abundance of bacteria, archaea, anaerobic ammonium oxidation (Anammox) bacteria and key genes (*amoA*, *nosZ*, *nirS* and *narG*) involved in nitrogen metabolism could affect the degradation of organic compounds and conversion of nitrogen in CWs [32,33].

For CI in the HSFCW and CA in VSFCW in our study, the microbial diversity was significantly higher in the RS than that in the NRS (*p* < 0.005). This finding was similar to previous reports in which the abundances of bacteria and total cells showed significantly higher values in the rhizosphere [17,34]. Our results showed that the microbial diversity of RS was similar that of NRS in two plants. Another study [35] also found significant differences in the microbial community but not in the microbial abundance among different plant species in constructed wetlands in summer. The low percentages of shared OTUs (Figure 4) indicated core microbes were obviously affected by two different plant roots, resulting in significant differences. Our results showed that the microbial diversity of CA was greater than that in CI, this is related to the larger underground biomass and denser roots of CA than those of CI.

### 4.2. Plant Effects on Microbial 16S rRNAs and Functional Genes

The copy numbers of the total bacterial 16S rRNAs and functional genes were reported to vary considerably with surroundings, sampling location and depth [36]. In the present study, their copy numbers were among the range of values that had been reported [36,37]. The functional genes of *hzsA* and AOB *amoA* in the sediment were higher in the RS compared with NRS in general, indicating that vegetation was positive for the ammonia nitrogen removal in the CWs. The primary influence of plant presence was believed to be related to ROL and its effect on rhizosphere redox [18] and the release of carbon compounds by plants [4]. Even if facultative anaerobic microbes (e.g., anammox bacteria harboring *hzsA* gene) prefer to gather in root surface for micro-aerobic environment, *E_h_* value of 0 was recommended for the single-stage partial nitritation/anammox process in the previous study [38]. More denitrifiers (harboring *narG*, *nirS* and *nosZ* genes) found in NRS than in RS might be due to the inhibition by ROL due to the susceptibility of denitrifying bacterial genes to oxygen in the denitrification pathway [39]. The copy numbers of *narG*, *nirS* and *nosZ* genes for CA were higher than those for CI, indicated CA had better denitrification ability and CI was more affected by ROL than CA. The total bacterial 16S rRNAs and AOB *amoA* had higher copy numbers of in the RS of CA than that of CI, which suggested more organic matter decomposers and ammonia oxidizers in the RS of CA. This may attribute to the significantly higher underground biomass and denser roots of CA than those of CI, which promoted the aerobes reproduction.

Our results showed that two species of plants had implications in multiple steps of the nitrogen cycle and could significantly (*p* < 0.05) alter the nitrogen removal microbes in CWs. Although CI and CA presented some differences in the effect on the functional genes, they had similar and positive effects on the degradation of organic compounds and ammonia oxidation process in general. However, the root exudates or certain plant species may have a direct impact on carbon and nitrogen cycling and may inhibit nitrification process by nitrifying microorganisms [8]. Some studies found wetland plants had negative or little influence on bacterial population or functional potential [7,18], in contrast to other studies [6,40]. Therefore, the biological inhibition for nitrogen removal could depend on different plant species and root exuded compound.

### 4.3. The contributions of Absorbing Nutrients and Heavy Metals by Two Plant Species

CI and CA are known for phytoremediation, their biomass affects the pollutant uptake capacity and the total removal capacity of CWs. The total root biomass can significantly influence the removal of ammonia and total dissolved phosphorus via processes such as plant uptake and nitrification [2]. The differences of nutrients in water and sediment were probably the main reason for the difference in the biomass of the same plant in different types of constructed wetlands (Tables S3 and S4). The uptake of NH_4_-N by plants required less energy than NO_3_-N and most of the large aquatic plants showed adaptation to use NH_4_-N as an inorganic nitrogen source [19]. This also can be proved by that NH_4_-N concentrations showed better correlations with the biomass than NO_3_-N (Table S6). In this study, the mixed supply of NO_3_-N and NH_4_-N could promote more growth of plants and produce higher absorb contributions more than a single supply of NH_4_-N or NO_3_-N [41,42]. No obvious harm on the growth of the two plant species was found as their biomass were comparable to those reported [2]. The allocation of nutrient and heavy metal in plant tissues in this study were similar to the previous reports [10,12]. In this study, we found that both of plants had stronger ability to accumulate Cu and Zn than other metals, which may be due to the uptake preferences to heavy metal [43]. CA had a good cumulative capacity for Cu and Zn and reached 75% and 6.72% of those in substrate [44], respectively. Our results confirmed that CI and CA tend to accumulate more heavy metals in roots than other tissues.

The contribution of plant absorption to pollutant removal can be estimated by comparing the pollutant removal load of the two treatment systems and the amount of pollutant absorbed by plants. The amount of pollutants absorbed by plants is proportional to the biomass and the concentration of pollutants in tissues. The difference of TN in different plant species was mainly related to plant species. In addition, it was positively related to the concentration of nutrient elements (e.g., TN) and biological abundance around root system. Microbes, such as ammonia oxidizing bacteria (AOB) and archaea (AOA) abundance were regulated by the levels of nitrogen, phosphorus and organic carbon in CWs [45]. While the results (Figure 5) also confirmed the copy numbers of the total bacterial 16S rRNAs, *narG*, *nirS* and *nosZ* genes for CA were higher than those for CI. As the front part of the whole HCW system, VSFCW removed more pollutants and the removal rates of organic pollutants and ammonia nitrogen were close to 60%. While HSFCW as the back part of the whole system, the removal rates of organic pollutants and ammonia nitrogen were 35% and 52%, respectively. The removal rate of nitrate nitrogen in HSFCW was higher than that in VSFCW. The pollutant removal loads of COD, ammonia nitrogen and TN in the whole wetland system were comparable with those of reported system [46], which has a hydraulic residence time of 10 days. Good combination of different wetland structures promoted the treatment performance. Obviously, VSFCW located in the front of the system undertook more pollutant removal than HSFCW. Based on the 150-day growth results in chapters 3.4 and 3.5, it can be seen that the contribution of plant absorption to the overall removal rate of wetland system can be neglected.

## 5. Conclusions

Wetland plants as the important component of constructed wetlands play a role in pollutants removal by affecting microbial communities rather than by absorbing pollutants. Results showed the presence of CI and CA positively affected microbial abundance and community in general and which was positive for the total bacteria and ammonia nitrogen removal in the CWs. Their root exudates may have a direct impact on nitrogen cycling, as they may inhibit nitrification process by soil nitrifying microorganisms. The root exudates of CA group had more strong inhibition compare to CI group. Some denitrifying bacteria may be inhibited by the ROL of root system. CA had better denitrification ability and CI was more affected by ROL than CA. Wetland plant species can significantly affect the distribution of microbial communities in RS. Plant selection is important to promote the development of microbial communities with a more active and diverse catabolic capability and the contribution of plant absorption to the overall removal rate of wetland system can be neglected. In the application of CWs, CI and CA should be planted at a more suitable density, which should be intensified when planted in VSFCW with main aim to remove organic matter and ammonia nitrogen. While in HSFCW with denitrification as its main function, planting should be reduced to meet only the necessary available carbon sources, especially for CI.

## Figures and Tables

**Table 1:** Biomass (g DW m^−2^) of two wetland plants grown at the end of 150 days in the VSFCW and HSFCW belong to the HCW system.

	Total	Roots	Rhizomes	Stem	Leaves	Flowers
CI						
V-CI1	703	60.3	201	196.3	231	14.4
V-CI2	686	57	193	193.4	227	15.6
H-CI3	599	45.8	168	176.5	198	10.7
H-CI4	576	41.5	156	177.7	191	9.8
*p*	<0.05	<0.05	ns	<0.05	<0.05	<0.05
CA						
V-CA1	665	117	248	275	20.9	4.1
V-CA2	689	122	252	288	22.7	4.3
H-CA3	540	89.7	214	216	16.9	3.4
H-CA4	527	87.4	209	212	15.4	3.2
*p*	<0.05	<0.05	<0.05	<0.05	<0.05	<0.05

Notes: DW represents dry weight; the samples numbers were shown in Figure 1.

## Supplementary Materials

The following are available online at https://www.mdpi.com/1660-4601/16/5/802/s1, Figure S1. α-Diversity comparison; Figure S2. The analysis chart of PCoA (Weighted Unifrac) for samples; Figure S3. Nutrients concentration, heavy metal concentration in rhizomes of the two plants at the eight sites in the HCW system. Table S1. Quantitative PCR primers and thermal cycling programs in this study; Table S2. Summary of 16S RNA Miseq sequences, operation taxonomic units (OTUs), and microbial diversity of sediment samples; Table S3. Averaged water quality monitoring data of a VSFCW and a HSFCW belonging to the HCW system; Table S4. Physicochemical properties of the rhizosphere sediment (RS) and non-rhizosphere sediment (NRS); Table S5. Nutrients concentration and heavy metal concentration in the leave and flower of two plants at the eight sites; Table S6. Pearson correlation coefficients between the plants biomass and physicochemical parameters of RS.
