# Supplementary material for: Impacting Microbial Communities and Absorbing Pollutants by Canna Indica and Cyperus Alternifolius in a Full-Scale Constructed Wetland System
Source: Int J Environ Res Public Health. 2019 Mar 5;16(5):802. doi: 10.3390/ijerph16050802 (PMC6427132; doi:10.3390/ijerph16050802)
Supplement: Supplementary file 1 [file ijerph-16-00802-s001.pdf]

Supplementary Material

Impacting Microbial Communities and Absorbing Pollutants by *Canna indica* and *Cyperus alternifolius* in a Full-scale Constructed Wetland System

Yinghai Wu [1,2], Tao He [3], Chen Chen [3], Xiaohang Fang [3], Dongyang Wei [3], Jing Yang [3], Renduo Zhang [2,*] and Rui Han [4,*]

[1] College of Marine and Civil Engineering, Dalian Ocean University, Dalian 116023, China; wuyinghai@dlou.edu.cn
[2] Guangdong Provincial Key Laboratory of Environmental Pollution Control and Remediation Technology, Sun Yat-sen University, Guangzhou 510275, China;
[3] South China Institute of Environmental Science, Ministry of Environment Protection, Guangzhou 510655, China; hetao@scies.org (T.H); chenchen7@scies.org (C.C.); fangxiaohang@scies.org (X.F.); weidongyang@scies.org (D.W.); yangjing@scies.org (J.Y.)
[4] College of Marine Technology and Environment, Dalian Ocean University, Dalian 116023, China
* Correspondence: Zhangrd@mail.sysu.edu.cn (R.Z.); hanrui@dlou.edu.cn (R.H.); Tel.: +86-20-84110052(R.Z); +86-411-84763257 (R.H.)

Detail Design of the Hybrid Wetland

The hybrid wetland (HCW) system receives sanitary and industrial mixed wastewater from a flat panel display factory. The wastewater was pretreated with a hydrolysis-acidification tank and aeration biofilm tank mechanically before flowing into the HCW system by gravity. After the tertiary treatment through the system, the water was discharged into Xinan River. HCW system was composed of a vertical subsurface flow constructed wetland (VSFCW), a free water surface (FWSCW), and a horizontal subsurface flow constructed wetland (HSFCW) in series, occupied an area of 5,000 m^2, in which areas of VSFCW, FWSCW, and HSFCW were 1,260, 1,250, and 2,490 m^2, respectively. Because of the limit of land area, the profiles of VSFCW and HSFCW were designed to a nearly rectangular shape, and that of FWSCW was trapezoid-shaped. The VSFCW consisted of three treatment units with equal area (each 18×23 m^2). The total depth of substrate gravel layer was 0.9 m. The substrate layer was divided into the upper, middle, and bottom layers with depths of 0.2, 0.5, and 0.2 m, respectively, and with gravel particle sizes of 25–40, 40–70, and 70–100 mm, respectively. The particle sizes were designed to be large enough to prevent the blockage problem under long operation and field conditions. *Canna indica* (CI) and *Cyperus alternifolius* (CA) were planted on the gravel layer of each unit. *Canna indica* was planted in the front of each unit with density of 0.9×0.9 m^2 and *Cyperus alternifolius* was planted in the back of each unit with density of 0.8×0.8 m^2, both of them were harvested with roots annually in the first half of January, June, and September. The FWSCW consisted of just one unit with different water depths. The surrounding section was with an area of 500 m^2 and water depth of 0.6 m, while the central section was with an area about 750 m^2 and water depth of 1.6 m. *Thalia dealbata*, *Pontederia cordata*, and *Cyperus papyrus* were planted densely in the shallow water area, and each plant species had an equal planting area with density of 0.3×0.3 m^2. *Elodea nuttallii*, *Ceratophyllum demersum L.*, and *Nymphaea alba* were planted sparsely in the deep water area, and each plant species had an equal planting area with density of 0.7×0.7 m^2. The HSFCW consisted of three treatment units with equal areas. The total depth of substrate layer was 0.9 m. Plant species were the same as those in the VSFCW. Both *Canna indica* and *Cyperus alternifolius* were harvested with roots when they grew to heights of 1.0 and 1.2 m, respectively, annually in the first half of January, June, and September. Geomembrane was installed under the wetland bottom to prevent exchange between wastewater and groundwater.

The height of drainage pipe outlet in each wetland was usually maintained the same as that of wetland fillers so that sewage could reach the same height of wetland filter and possibly filled the whole porous volume to make full use of the function of microorganisms in the layer. A catchment ditch and buried pipeline were used to connect the VSFCW and FWSCW, and a buried pipeline was used to connect the FWSCW and HSFCW.

A detail of the sampling procedure

The sediment remained attached to plant roots was carefully stripped and collected as the RS sample in each location. Thus total 16 sediment samples were collected. The samples were stored in bags and placed on ice immediately after sampling. In the laboratory, the samples were homogenised manually and visible root or plant materials were manually removed. Then the samples were stored at -80℃ before use. Water samples were collected at the inlets and outlets of the VSFCW and HSFCW, respectively. All water samples were transferred immediately to the lab and stored at 4℃ before analysis.

DNA extraction

Genomic DNAs of the sediment samples were extracted using the MO-BIO PowerSoil® DNA Isolation Kit (MO BIO Laboratories, Inc., Carlsbad, USA) following the manufacturer's directions. Extracted DNA concentrations were determined through Nanodrop Spectrophotometer ND-1000 (Thermo Fisher Scientific, USA). Additionally, extracted genomic DNA was detected by 1.5% agarose gel electrophoresis and stored at -20℃ until use. The DNA concentration was determined using a NanoDrop (NanoDrop Technologies Inc., Wilmington, DE, USA) and Pico Green assays.

Quantitative real-time polymerase chain reaction

The 50 μL PCR mixture contained 0.25 μL 5U μL^{-1} *Ex Taq* DNA polymerase (TaKaRa, Dalian, China), 5 μL 10 × *Ex Taq* buffer (TaKaRa, Dalian, China), 4 μL dNTP Mixture (2.5 mM each) (TaKaRa, Dalian, China), 1 μL 10 μM primer pairs, 1 μL template DNA. PCR product sizes were determined by 1.2% agarose gel electrophoresis at constant voltage. PCR amplification products were pooled and purified by E.Z.N.A.® Gel Extraction Kit (Omega Bio-Tek, Norcross, GA, USA) following the manufacturer's instruction. The specific gene fragments amplified from environmental samples were connected to pGEM-T easy Vector (Promaga corporation USA), and cloned into *E.coli* DH 5*α* competent cells (TaKaRa clontech China). Then, the strains were screened on ampicillin (50 mg L^{-1}) plates and incubated at 37℃ overnight, blue-white selection was used for colony/stain selection; the white strains were selected as positive suspicious strains, further selected via colony PCR, and the obtained strains were subsequently sent to BGI Inc. (Shenzhen, China) for sequencing. Sequencing results were used for BLAST homology analysis. Recombinant plasmids were extracted and purified using E.Z.N.A.® Plasmid Mini Kit I (Omega Bio-Tek, Norcross, GA, USA). The plasmid DNAs were quantified by Qubit® 2.0 Fluorometer (Invitrogen, Carlsbad, CA), and were diluted to yield a series of concentrations, each with 10-fold differences. The standard plasmids for total bacteria 16S rRNAs, *hzsA*, AOB amoA, *narG*, *nirS* and *nosZ* genes were prepared in the range of 1.27×10^{8}-1.27×10^{12} copies, 7.55×10^{4}-7.55×10^{9} copies, 6.83×10^{4}-6.83×10^{9} copies, 8.56×10^{4}-8.56×10^{9} copies, 2.62×10^{4}-2.62×10^{9} copies, 3.54×10^{4}-3.54×10^{9} copies, respectively. Each R^2-value of each standard curve for each replicate exceeded 0.99. Both the standard plasmid DNAs and the samples were added into 96-well plates, and were carried out in triplicate. The 20 μl reaction mixtures contain 10 μl SYBR Green supermix (Bio-Rad, Laboratories, Hercules, CA), 0.2 μl primer pairs, 1 μl DNA template DNA.

PCR amplification

The 30 μL PCR mixture contained 0.75 units *Ex Taq* DNA polymerase (TaKaRa, Dalian, China), 1 × *Ex Taq* loading buffer (TaKaRa, Dalian, China), 0.2 mM dNTP mix (TaKaRa, Dalian, China), 0.2 μM of each primer, 50 ng template DNA. The PCR was performed at 94 °C for 5 min, 30 cycles of 94

°C for 30 s, 53 °C for 1 min, and 72 °C for 1 min, and a final extension at 72 °C for 7 min using the AccuPrime High Fidelity *Taq* Polymerase (Invitrogen, Grand Island, NY, USA). The PCR products were pooled and purified using the E.Z.N.A.® Gel Extraction Kit (Omega Bio-Tek, Norcross, GA, USA).

High-throughput sequencing data analysis

The 16S rRNA MiSeq sequencing data were processed and analyzed with the modified pipelines of mothur and UPARSE [1,2]. As described by [3], reads with a quality less than 30 at the 3′ end were trimmed. The command "make.contigs" was then used to combine the two sets of reads for each sample. Next, the commands "screen.seqs", "unique.seqs", "align.seqs", "filter.seqs", "pre.cluster", and "chimera.uchime" in the mother platform were used to reduce sequencing error and identify and remove putative chimeras. Finally, the quality sequences were clustered into operational taxonomic units (OTUs) at the 97% similarity level using the "cluster_otus" command in the UPARSE platform. Representative sequences were aligned and then used to build the neighbor-joining phylogenetic tree with FastTree [4]. Taxonomic assignment was determined at the 80% threshold with the RDP Classifier (version 2.6) [5].

Physicochemical analyses

Measured physicochemical properties of water included pH, dissolved oxygen (DO), redox potential (E_h), chemical oxygen demand (COD), NH_4-N, NO_3-N, TN, TP, Cd, Cu, Ni, and Zn. pH, DO, and E_h were measured in situ using a portable multi-parameter monitor (HACH sensION+MM150, USA). Measurements were repeated three times at each CW. COD, NH_4-N, TN, and TP were measured with the dichromate titration method, the Nessler's reagent spectrometric method, the alkaline potassium persulfate digestion UV spectrophotometric method, and the ammonium molybdate spectrophotometric method, respectively [6]. NO_3-N was measured with the ultraviolet spectrophotometric method [7]. Cd, Cu, Ni, and Zn were measured using an ICP-6300 inductively coupled plasma-atomic emission spectrometer (Thermo Scientific, USA).

Sediment samples were used to measure pH, E_h, total organic carbon (TOC), NH_4-N, NO_3-N, TN, TP, Cd, Cu, Ni and Zn. E_h was measured in situ as above. The sediment samples were air-dried and homogenised. TOC was measured with a TOC analyzer (TOC-V CPH, Shimadzu, Japan). Sediment samples for NH_4-N and NO_3-N analysis were homogenized and frozen as soon as possible. Sediment samples for nitrogen analysis were extracted with 1 M KCl [8]. Subsequent analyses of NH_4-N and NO_3-N were carried out using continuous-flow analyzer (Flow Solution IV, OI, USA) [9]. TN was measured by Kjeldahl digestion procedures [10]. TP was determined by the alkali fusion–Mo-Sb Anti-spectrophotometric method [11]. Sediment samples were digestion with a $HClO_4$:HNO_3:HCl (7:2:5) mixture. The result solution were analyzed for Cd, Cu, Ni, Zn by ICP-6300 inductively coupled plasma-atomic emission spectrometer (Thermo Scientific, USA).

TN concentration in plant was determined after digestion of powdered dry shoot material (500 mg) in a Digesdahl flask (HACH) with 4 ml H_2SO_4 (98% v/v) and 10 ml H_2O_2 (40% v/v), brought to a final volume of 100 ml with deionised water and adjusted to neutral pH with 6 M NaOH. After addition of 2 ml of Nessler's reagent the absorbance was read at 425 nm using a spectrophotometer [12]. The TP was determined by digesting plant samples with sulphuric-nitric acid followed by the vanadomolybdophosphoric acid colorimetric method [12]. Cd, Cu, Ni and Zn in above and belowground parts were determined in the same way as in the sediment samples.

Statistical Analyses

All statistical analyses were implemented using SPSS 18.0 software. An independent sample t-test was used to examine the difference between the biomass of same plant species in different types of CWs. Pearson correlation coefficients were calculated to evaluate the relationships between biomass and physicochemical parameters. One-way analysis of variance (ANOVA) was

performed to compare differences in physicochemical properties of water among the sampling sites, as well as physicochemical properties of sediment, nutrients concentration, heavy metal concentration, and functional genes numbers. Post-hoc tests with Duncan's statistics at $p = 0.05$ was performed to analyze the differences between groups of data.

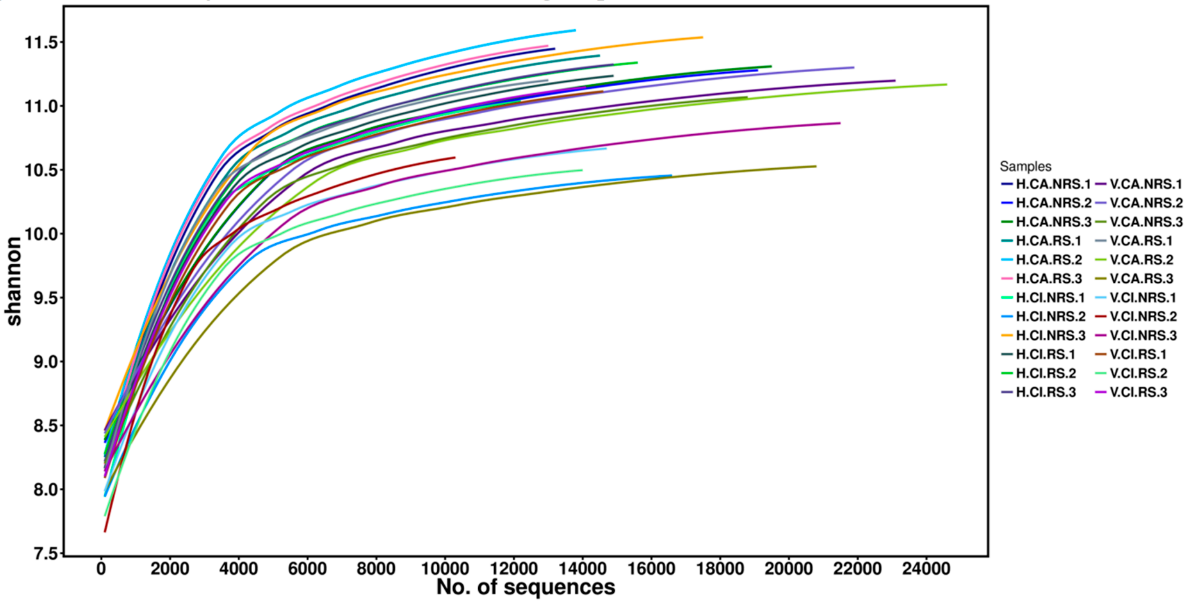

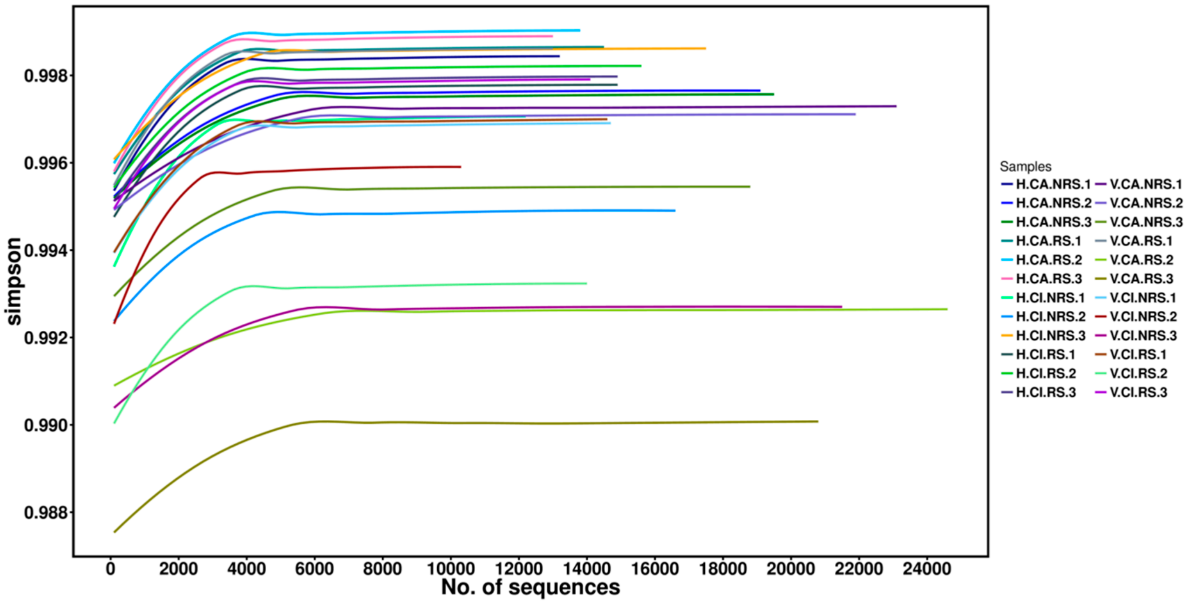

Figure S1. α-Diversity comparison. Rarefaction curves for Shannon index (a) and Simpson index (b) were calculated using Mothur (v1.27.0) with reads normalized to 10,335 for each sample using 0.03 distance OTUs. The sediment samples were collected at depths of 20 cm in the VSFCW and HSFCW for both rhizosphere sediment (RS) samples and non-rhizosphere sediment (NRS) samples. The three samples in each zone were numbered with 1, 2, and 3.

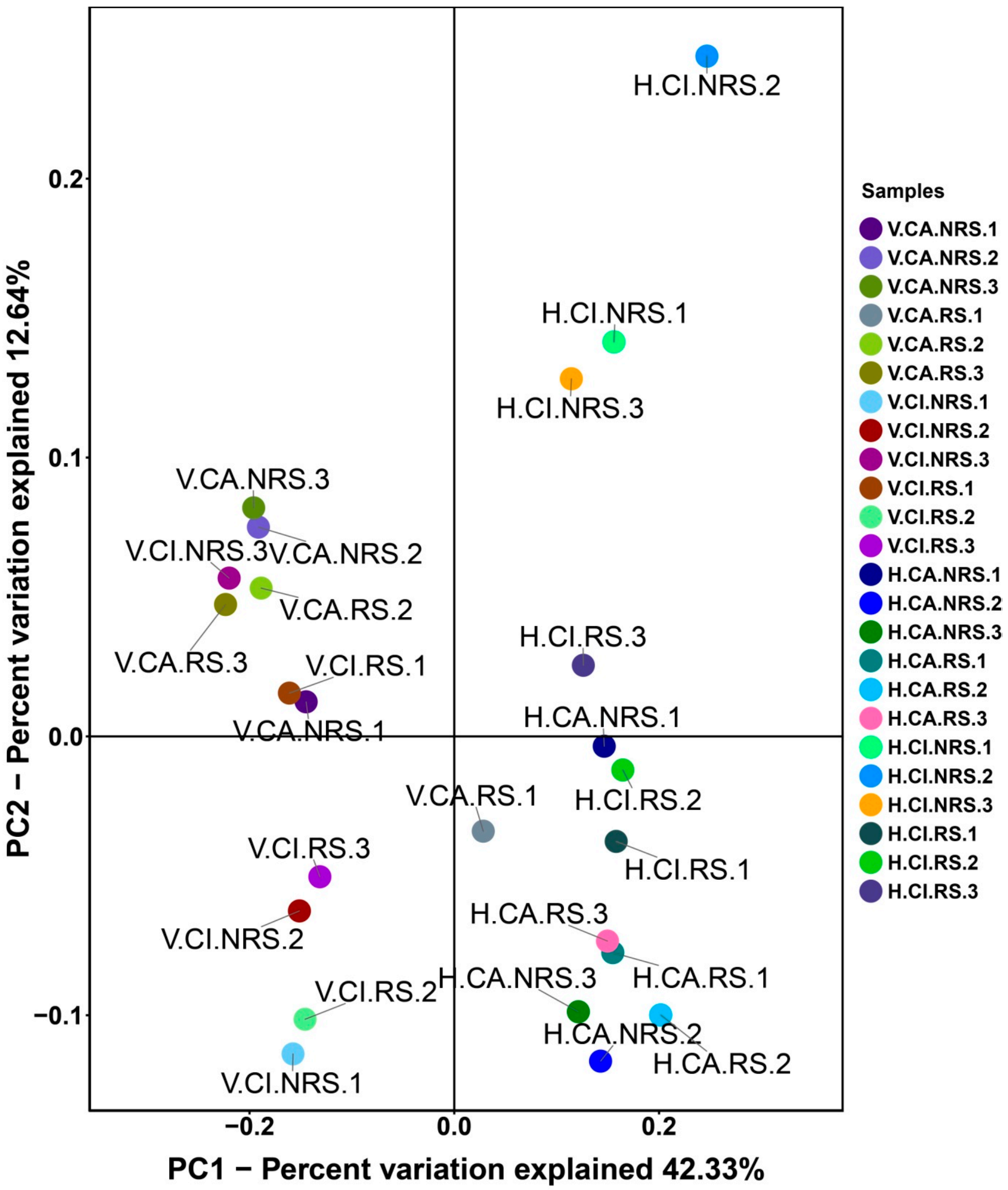

Figure S2. The analysis chart of PCoA (Weighted Unifrac) for samples. In the sample number: V = VSFCW; H = HSFCW; CI = *Canna indica*; CA = *Cyperus alternifolius*; RS = rhizosphere; NRS = non-rhizosphere; the three duplicates in each zone were numbered with 1, 2, and 3.

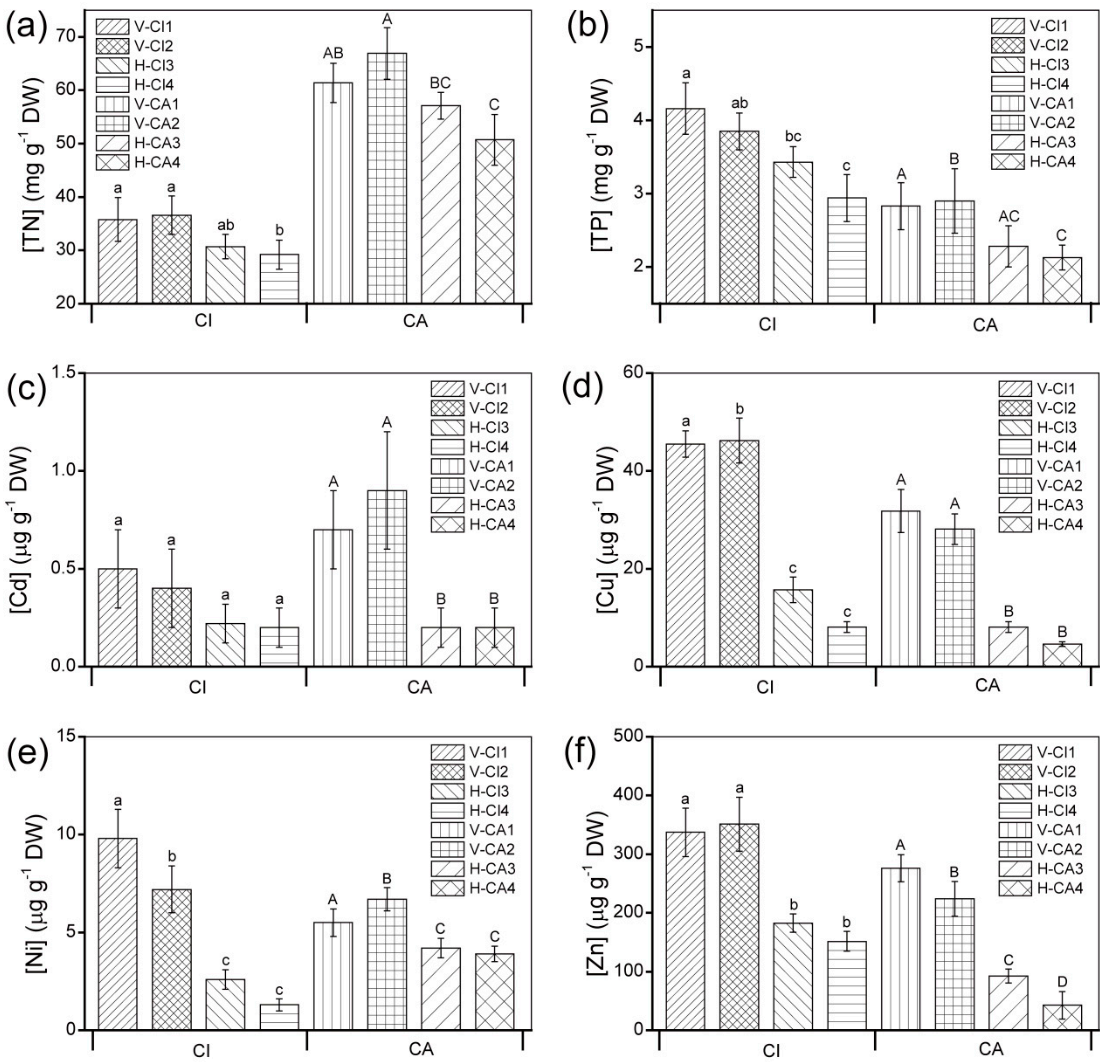

Figure S3. Nutrients concentration (mg g-1 DW), heavy metal concentration (μg g-1 DW) in rhizomes of the two plants at the eight sites in the HCW system. (a) TN; (b) TP; (c) Cd; (d) Cu; (e) Ni; (f) Zn. The samples numbers were shown in Figure 1.

Table S1. Quantitative PCR primers and thermal cycling programs in this study.

Target prokaryote	Target gene	Oligonucleotide sequence (5′-3′) of primer pairs	Annealing (℃)	Thermal cycling programs	Reference
Total bacteria	16S rRNA	338F: CCTACGGGAGGCAGCAG<br>518R: ATTACCGCGGCTGCTGG	55	a 94℃ pre-denaturing for 5 min, 39 cycles of a 94℃ denaturing for 15 s, a 55℃ annealing for 1 min, and finally a 72℃ extension for 10 min	[13]
Anammox Bacteria	hzsA	hzsA 1597F: WTYGGKTATCARTATGTAG<br>hzsA 1857R: AAABGGYGAATCATARTGGC	55	a denaturation step of 5 min at 96℃, followed by 30 cycles of denaturation (1 min at 96℃), primer annealing (1 min), and extension (1.5 min at 72℃), and finally a last extension step of 5 min at 72℃	[14]
AOB	*amoA*	amoA-1F: GGGGTTTCTACTGGTGGT<br>amoA-2R: CCCCTCKGSAAAGCCTTCTTC	57	5 min at 94℃, followed by 40 cycles of 30 s at 94℃, 1 min at 57℃, and 10 min at 72℃	[15]
Denitrifying bacteria	*nirS*	cd3aF : AACGYSAAGGARACSGG<br>R3cd: GASTTCGGRTGSGTCTTSAYGAA	51/72	an initial denaturation of the DNA at 94℃ for 2 min, followed by 35 cycles of 30 s at 94℃, 1 min at 51℃ and 1 min at 72℃; and 10 min at 72℃	[16]
Denitrifying bacteria	*nosZ*	F: CGYTGTTCMTCGACAGCCAG<br>1622R: CGSACCTTSTTGCCSTYGCG	58/53	a 94 ℃ pre-denaturing for 2 min, a 94℃ denaturing for 30 s, a 58℃ annealing for 30 s, a 72℃ extension for 30 s, the temperature was decreased by 0.5℃ every second cycle for first 10 cycles, and 25 cycles of a 53℃ annealing; and finally a 72℃ extension for 10 min	[17]
Dissimilarity nitrite reducing bacteria	*narG*	1960m2F: TAYGTSGGGCAGGARAAACTG<br>2050m2R: CGTAGAAGAAGCTGGTGCTGTT	55	a 94℃ pre-denaturing for 5 min, 8 cycles of a 94℃ denaturing for 30 s, a 63℃ annealing for 30 s, and a 72℃ extension for 30 s; 35 cycles of a 94℃ denaturing for 30 s, a 57 ℃ annealing for 30 s, a 72℃ extension for 30 s; and finally a 72℃ extension for 10 min	[18]

Table 2. Summary of 16S RNA Miseq sequences, operation taxonomic units (OTUs), and microbial diversity of sediment samples, which were collected at depths of 20 cm in the VSFCW and HSFCW for both rhizosphere sediment (RS) samples and non-rhizosphere sediment (NRS) samples. The three samples in each zone were numbered with 1, 2, and 3.

Sample ID	No. of sequences	No. of OTUs	Chao1	Observed_species	PD_whole_tree	Shannon	Smpson
H.CA.NRS.1	13278	6148	16513.38	5118	349	11.30553	0.998412
H.CA.NRS.2	19130	7347	14881.08	4726	333	10.96362	0.997647
H.CA.NRS.3	19577	7488	14745.58	4746	328	10.98027	0.997539
H.CA.RS.1	14503	6241	15008.99	4890	332	11.20991	0.998625
H.CA.RS.2	13808	6352	16279.56	5145	351	11.42292	0.999003
H.CA.RS.3	13057	5994	15518.14	5058	341	11.34065	0.998881

H.CI.NRS.1	12225	5195	13926.11	4604	311	10.95346	0.997104
H.CI.NRS.2	16651	5243	9878.164	3795	269	10.25201	0.99483
H.CI.NRS.3	17599	6991	13848.97	4827	321	11.25391	0.998574
H.CI.RS.1	14931	6238	14816.59	4767	320	11.02993	0.997732
H.CI.RS.2	15631	6420	14595.43	4771	322	11.11744	0.998188
H.CI.RS.3	14912	6341	15670.28	4849	323	11.12403	0.99795
V.CA.NRS.1	23134	7961	13481.23	4545	283	10.81315	0.997243
V.CA.NRS.2	21983	8151	14786.39	4763	290	10.91004	0.997061
V.CA.NRS.3	18812	7118	14445.63	4613	283	10.74936	0.995309
V.CA.RS.1	13015	5302	12637.34	4516	312	11.0828	0.998589
V.CA.RS.2	24652	8624	14737.44	4675	297	10.736	0.9926
V.CA.RS.3	20813	6648	11533.97	4109	269	10.22573	0.990184
V.CI.NRS.1	14750	5238	12094.5	4069	269	10.50087	0.996854
V.CI.NRS.2	10335	4325	13767.31	4325	283	10.6044	0.995907
V.CI.NRS.3	21559	7612	14261.43	4480	278	10.50458	0.992667
V.CI.RS.1	14616	6058	14850.54	4701	295	10.92467	0.99693
V.CI.RS.2	14022	4953	10158.87	4019	272	10.3571	0.99317
V.CI.RS.3	14188	6021	15976.51	4751	308	10.97075	0.997872

Microbial alpha diversity (e.g., Chao1, Shannon and Smpson) was estimated based on 97% OTU (operational taxonomic units) clusters according to a subset of 10,335 randomly selected effective sequences per sample with 100 iterations.

Table S3. Averaged water quality monitoring data of a VSFCW and a HSFCW belonging to the HCW system.

Parameters	VSFCW		HSFCW		*p*
	Inlet	Outlet	Inlet	Outlet	
Temperature (°C)	26.6 ± 0.2 a	25.5 ± 0.2 b	27.1 ± 0.1 c	26.0 ± 0.2 d	<0.001
DO (mg L^{-1})	6.75 ± 0.3 a	0.80 ± 0.25 c	3.70 ± 0.4 b	0.42 ± 0.1 c	<0.001
pH	7.27 ± 0.24	7.16 ± 0.2	7.11 ± 0.17	7.25 ± 0.25	ns
E_h (mV)	132 ± 17 a	-218 ± 13 c	18 ± 9 b	-234 ± 11 c	<0.001
COD (mg L^{-1})	52.8 ± 5.6 a	21.7 ± 4.2 bc	23.5 ± 3.4 b	15.3 ± 2.7 c	<0.001
NH_4-N (mg L^{-1})	8.25 ± 1.25 a	3.5 ± 0.28 b	3.27 ± 0.19 b	1.54 ± 0.2 c	<0.001
NO_3-N (mg L^{-1})	9.1 ± 2.22 a	9.46 ± 1.83 a	8.19 ± 2.1 a	3.72 ± 0.85 b	<0.05
TN (mg L^{-1})	18.4 ± 1.9 a	14.36 ± 2.43 ab	13.67 ± 2.21b	6.37 ± 2.13 c	<0.05
TP (mg L^{-1})	1.32 ± 0.2	0.9 ± 0.26	1.12 ± 0.25	0.85 ± 0.22	ns
Cd (mg L^{-1})	0.003 ± 0.002	0.002 ± 0.001	0.001 ± 0.001	<0.001	ns
Cu (mg L^{-1})	0.013 ± 0.003 a	0.009 ± 0.003 bc	0.007 ± 0.001 c	0.005 ± 0.002 c	<0.05
Ni (mg L^{-1})	0.015 ± 0.002 a	0.012 ± 0.001 bc	0.011 ± 0.003 c	0.01 ± 0.002 c	<0.05

Zn (mg L^{-1})	0.015 ± 0.003	0.012 ± 0.002	0.01 ± 0.002	0.009 ± 0.001	ns

Notes: a) VSFCW represent vertical subsurface flow constructed wetland; b) HSFCW represent horizontal subsurface flow constructed wetland; c) ICW represent hybrid constructed wetland; d) average and standard deviations values were calculated from three repeats (mean ± SD,n = 3) for each sample.

Table S4. Physicochemical properties of the rhizosphere sediment (RS) and non-rhizosphere sediment (NRS).

**Sample**	**pH**	E_h (mV)	**TOC** (%)	NH_4^+-N (mg/kg)	NO_3^--N (mg/kg)	**TN** (g/kg)	**TP** (g/kg)
CI							
V-CI1-RS	6.97 ± 0.12 ab	-96 ± 8 d	5.64 ± 0.45 c	103.3 ± 5.9 d	112.3 ± 8.7 cd	1.84 ± 0.15 cd	1.36 ± 0.10 abc
V-CI1-NRS	7.19 ± 0.13 cd	-89 ± 11 d	4.25 ± 0.50 b	85.9 ± 11.0 c	105.8 ± 8.1 bc	1.94 ± 0.20 d	1.31 ± 0.15 abc
V-CI2-RS	7.13 ± 0.09 bcd	-92 ± 20 d	6.07 ± 0.71 c	118.2 ± 4.2 e	96.7 ± 4.6 b	1.81 ± 0.15 cd	1.55 ± 0.12 a
V-CI2-NRS	7.34 ± 0.09 d	-110 ± 22 cd	4.18 ± 0.39 b	82.6 ± 8.6 c	104.6 ± 9.3 bc	1.77 ± 0.13 bcd	1.46 ± 0.19 ab
H-CI3-RS	6.89 ± 0.14 a	-136 ± 18 bc	3.27 ± 0.51 ba	47.8 ± 10.8 ab	120.2 ± 5.4 de	1.63 ± 0.17 abc	1.21 ± 0.20 bc
H-CI3-NRS	7.12 ± 0.06 bc	-165 ± 9 ab	2.68 ± 0.42 a	55.3 ± 6.4 b	132.5 ± 8.2 de	1.52 ± 0.08 ab	1.20 ± 0.09 bc
H-CI4-RS	7.15 ± 0.13 bcd	-177 ± 18 a	2.90 ± 0.78 a	40.5 ± 8.9 a	93.4 ± 6.1 b	1.48 ± 0.07 a	1.13 ± 0.10 c
H-CI4-NRS	7.20 ± 0.11 cd	-180 ± 23 a	2.71 ± 0.66 a	47.7 ± 4.1 ab	71.1 ± 11.2 a	1.50 ± 0.13 a	1.16 ± 0.10 c
P	<0.05	<0.001	<0.001	<0.001	<0.001	<0.05	<0.05
CA							
V-CA1-RS	7.04 ± 0.10 abc	-86 ± 17 c	6.06 ± 0.85 c	96.5 ± 7.3 b	94.9 ± 8.5 a	1.62 ± 0.07 bc	1.28 ± 0.2 bc
V-CA1-NRS	7.16 ± 0.09 cd	-105 ± 20 bc	5.70 ± 0.23 cb	120.6 ± 9.8 c	101.5 ± 6.1 a	1.80 ± 0.05 c	1.39 ± 0.1 c
V-CA2-RS	7.11 ± 0.08 bcd	-93 ± 15 c	5.81 ± 0.32 cb	94.3 ± 10.4 b	118.7 ± 10.5 b	1.76 ± 0.10 bc	1.42 ± 0.12 c
V-CA2-NRS	7.28 ± 0.05 d	-92 ± 22 c	5.12 ± 0.56 b	105.7 ± 8.1 b	98.4 ± 9.4 a	1.75 ± 0.13 bc	1.34 ± 0.3 bc
H-CA3-RS	6.86 ± 0.13 ab	-126 ± 14 b	3.55 ± 0.24 a	51.1 ± 3.5 a	104.0 ± 5 a	1.58 ± 0.09 bc	1.17 ± 0.1 abc
H-CA3-NRS	6.95 ± 0.14 ab	-133 ± 13 b	3.13 ± 0.51 a	40.0 ± 9.6 a	125.5 ± 7.3 b	1.61 ± 0.15 bc	1.05 ± 0.1 ab
H-CA4-RS	7.02 ± 0.08 ac	-167 ± 18 a	3.89 ± 0.45 a	45.7 ± 4.8 a	103.5 ± 9.0 a	1.37 ± 0.14 a	1.04 ± 0.2 ab
H-CA4-NRS	7.15 ± 0.10 cd	-174 ± 9 a	3.30 ± 0.30 a	40.6 ± 6.2 a	91.8 ± 5.3 a	1.55 ± 0.20 ab	0.95 ± 0.1 a
P	<0.05	<0.001	<0.001	<0.001	<0.05	<0.05	<0.05

**Sample**	**Cd** (mg/kg)	**Cu** (mg/kg)	**Ni** (mg/kg)	**Zn** (mg/kg)
CI				
V-CI1-RS	2.0 ± 0.3 cd	197.5 ± 9.4 a	36.6 ± 2.1 bc	1254.6 ± 15.8 d
V-CI1-NRS	2.4 ± 0.4 d	184.4 ± 7.3 ab	43.5 ± 4.0 d	1190.5 ± 20.6 c
V-CI2-RS	1.8 ± 0.1 c	186.5 ± 10.2 ab	38.8 ± 3.3 cd	1178.8 ± 43.1 c
V-CI2-NRS	2.1 ± 0.2 cd	178.6 ± 13.5 b	31.3 ± 5.1 b	1243.8 ± 22.2 d
H-CI3-RS	0.5 ± 0.1 a	53.8 ± 4.0 c	23.4 ± 2.5 a	470.1 ± 41.7 a
H-CI3-NRS	1.2 ± 0.2 b	46.1 ± 6.5 c	20.8 ± 3.6 a	490.9 ± 35.3 ab

H-CI4-RS	0.8 ± 0.1 ab	34.6 ± 4.3 d	20.3 ± 2.7 a	531.3 ± 18.5 b
H-CI4-NRS	1.1 ± 0.3 b	54.2 ± 4.6 cd	22.4 ± 1.9 a	510.6 ± 23.3 ab
P	<0.001	<0.001	<0.001	<0.001
CA				
V-CA1-RS	1.8 ± 0.1 cd	207.9 ± 12.0 d	38.1 ± 2.2 d	1260.2 ± 26.7 d
V-CA1-NRS	1.6 ± 0.1 c	194.7 ± 8.8 cd	33.4 ± 2.5 c	1210.0 ± 45.5 c
V-CA2-RS	1.7 ± 0.2 cd	186.4 ± 11.4 c	28.9 ± 1.9 cb	1194.7 ± 35.5 c
V-CA2-NRS	2.0 ± 0.2 d	196.8 ± 9.5 cd	30.5 ± 3.5 c	1285.0 ± 17.5 d
H-CA3-RS	1.1 ± 0.1 b	61.3 ± 6.7 b	23.6 ± 2.7 a	551.5 ± 28.9 b
H-CA3-NRS	0.7 ± 0.3 a	70.5 ± 5.1 b	24.8 ± 1.7 ba	590.4 ± 31.4 b
H-CA4-RS	0.9 ± 0.1 ab	44.7 ± 5.6 a	27.7 ± 3.4 cb	452.1 ± 12.8 a
H-CA4-NRS	1.0 ± 0.2 ab	56.4 ± 3.7 ab	21.0 ± 2.6 a	564.7 ± 18.1 b
P	<0.001	<0.001	<0.001	<0.001

Notes: a) V and H represent the VSFCW and HSFCW, respectively; b) CI and CA represent *Canna indica* and *Cyperus alternifolius*, respectively; c) the numbers of plant samples were shown in Fig 1; d) average and standard deviations values were calculated from three repeats (mean ± SD, n=3) for each sample.

Table S5. Nutrients concentration (mg g^{-1} DW) and heavy metal concentration (μg g^{-1} DW) in the leave and flower of two plants at the eight sites.

	**CI**					**CA**				
	**V-CI1**	**V-CI2**	**H-CI3**	**H-CI4**	***P***	**V-CA1**	V-CA2	H-CA3	H-CA4	*P*
Leaves										
TN	27.5 ± 4.2 a	25.6 ± 3.33 a	23.4 ± 3.5 a	22.3 ± 2.64 a	ns	45.6 ± 3.64 A	48.8 ± 5.27 A	34.3 ± 6.18 B	29.5 ± 4.81 B	<0.05
TP	2.11 ± 0.79 a	1.75 ± 0.55 ab	1.26 ± 0.43 ab	0.88 ± 0.32 b	ns	1.18 ± 0.35 A	1.25 ± 0.41 A	0.86 ± 0.12 AB	0.53 ± 0.18 B	ns
Cd	<0.1 a	<0.1 a	<0.1 a	<0.1 a	ns	<0.1 A	<0.1 A	<0.1 A	<0.1 A	ns
Cu	5.7 ± 0.4 a	4.6 ± 0.5 b	2.6 ± 0.2 c	1.2 ± 0.2 d	<0.001	3.1 ± 0.5 A	3.4 ± 0.6 A	1.6 ± 0.5 B	1.4 ± 0.4 B	<0.05
Ni	0.3 ± 0.1 a	<0.1 b	<0.1 b	<0.1 b	<0.001	<0.1 A	0.2 ± 0.1 B	<0.1 A	<0.1 A	<0.001
Zn	23.1 ± 3.2 a	18.9 ± 2.2 b	11.4 ± 1.9 c	5.7 ± 0.4 d	<0.001	17.5 ± 3.1 A	22.1 ± 2.6 B	9.8 ± 1.1 C	6.6 ± 0.9 C	<0.001
Flowers										
TN	22.3 ± 4.5 a	23.6 ± 3.1 a	22.6 ± 3.9 a	20.9 ± 2.2 a	ns	31.3 ± 4.38 AB	37.8 ± 6.52 A	23.6 ± 5.17 BC	20.9 ± 3.61 C	<0.05
TP	4.74 ± 0.71 a	3.79 ± 0.66 ab	3.97 ± 0.2 ab	3.41 ± 0.33 b	ns	4.54 ± 0.46 A	4.68 ± 0.42 A	3.82 ± 0.28 B	3.20 ± 0.34 B	<0.05
Cd	<0.1 a	<0.1 a	<0.1 a	<0.1 a	ns	<0.1 A	<0.1 A	<0.1 A	<0.1 A	ns
Cu	7.3 ± 1.1 a	6.5 ± 1.5 ab	4.7 ± 0.9 bc	2.8 ± 0.5 c	<0.05	4.2 ± 0.5 A	5.3 ± 0.3 B	3.4 ± 0.5 AC	2.7 ± 0.4 C	<0.001
Ni	<0.1 a	<0.1 a	<0.1 a	<0.1 a	ns	<0.1 A	<0.1 A	<0.1 A	<0.1 A	ns
Zn	31.4 ± 2.6 a	39.7 ± 3.4 b	23.4 ± 2.8 c	18.9 ± 1.9 c	<0.001	21.3 ± 2.1 A	17.8 ± 2.6 A	13.7 ± 1.7 B	11.7 ± 2.0 B	<0.05

Table S6. Pearson correlation coefficients between the plants biomass (g DW m^{-2}) and physicochemical parameters of RS.

		NH_4-N	NO_3-N	TN	TP	Cd	Cu	Ni	Zn
CI	Belowground biomass	0.94	0.17	0.99*	0.83	0.92	0.99*	0.96*	0.96*
	Aboveground biomass	0.97*	0.00	0.96*	0.87	0.97*	1.00**	0.98*	0.99*
CA	Belowground biomass	0.99**	0.27	0.84	0.93	0.98*	0.98*	0.66	0.99*
	Aboveground biomass	0.98*	0.32	0.84	0.93	0.96*	0.97*	0.63	0.98*

** Statistically significant (P < 0.01, two-sided test). * Statistically significant ($p < 0.05$, two-sided test).

References

1. Edgar RC (2013) UPARSE: highly accurate OTU sequences from microbial amplicon reads. Nat Methods, 10:996–998. doi.org/10.1038/nmeth.2604.
2. Kozich JJ, Westcott SL, Baxter NT, Highlander SK, Schloss PD (2013) Development of a dual-index sequencing strategy and curation pipeline for analyzing amplicon sequence data on the MiSeq Illumina sequencing platform. Appl. Environ. Microbiol., 79:5112–5120. doi.org/10.1128/aem.01043-13.
3. Liu J, He XX, Lin XR, Chen WC, Zhou QX, Shu WS, Huang LN (2015) Ecological effects of combined pollution associated with e-waste recycling on the composition and diversity of soil microbial communities. Environ. Sci. Technol., 49:6438–6447. doi.org/10.1021/es5049804.
4. Price MN, Dehal PS, Arkin AP (2009) FastTree: computing large minimum evolution trees with profiles instead of a distance matrix. Mol. Biol. Evol., 26:1641–1650. doi.org/10.1093/molbev/msp077.
5. Qiong W, Garrity GM, Tiedje JM, Cole JR (2007) Naive Bayesian classifier for rapid assignment of rRNA sequences into the new bacterial taxonomy. Appl. Environ. Microbiol., 73:5261–5267. doi.org/10.1128/aem.00062-07.
6. Wang W, Gao J, Guo X, Li W, Tian X, Zhang R. Long-term effects and performance of two-stage baffled surface flow constructed wetland treating polluted river. Ecol. Eng., 2012, 49: 93-103. doi.org/10.1016/j.ecoleng.2012.08.016.
7. Lu J, Fu Z, Yin Z. Performance of a water hyacinth (*Eichhornia crassipes*) system in the treatment of wastewater from a duck farm and the effects of using water hyacinth as duck feed. J. Environ. Sci., 2008, 20(5): 513-519. doi.org/10.1016/s1001-0742(08)62088-4.
8. Kachurina O M, Zhang H, Raun W R, Krenzer E G. Simultaneous determination of soil aluminum, ammonium- and nitrate-nitrogen using 1M potassium chloride extraction. Commun. .Soil Sci. Plan., 2000, 31(7-8): 893-903. doi.org/10.1080/00103620009370485.
9. Lopez Pasquali, C E, Fernandez Hernando P, Durand Alegria J S. Spectrophotometric simultaneous determination of nitrite, nitrate and ammonium in soils by flow injection analysis. Anal. Chim. Acta., 2007, 600(1-2): 177-182. doi.org/10.1016/j.aca.2007.03.015.
10. Chen Y T, Li J T, Chen L X, Hua Z S, Huang L N, Liu J, Xu B B, Liao B, Shu W S. Biogeochemical processes governing natural pyrite oxidation and release of acid metalliferous drainage. Environ. Sci. Technol., 2014, 48(10): 5537-5545. doi.org/10.1021/es500154z.
11. Jie W, Liu X, Cai B. Diversity of rhizosphere soil arbuscular mycorrhizal fungi in various soybean cultivars under different continuous cropping regimes. PLoS One, 2013, 8(8): e72898. doi.org/10.1371/journal.pone.0072898.
12. Torit J, Siangdung W, Thiravetyan P. Phosphorus removal from domestic wastewater by *Echinodorus cordifolius L.* J. environ. Sci. heal. A., 2012, 47(5): 794-800. doi.org/10.1080/10934529.2012.660114.
13. Muyzer G, De Waal E C, Uitterlinden, A G. Profiling of complex microbial populations by denaturing gradient gel electrophoresis analysis of polymerase chain reaction-amplified genes coding for 16S rRNA. Appl. Environ. Microbiol., 1993, 59(3): 695-700.
14. Harhangi H R, Le Roy M, van Alen T, Hu B L, Groen J, Kartal B, Tringe S G, Quan Z X, Jetten M S, Op den Camp H J. Hydrazine synthase, a unique phylomarker with which to study the presence and biodiversity of anammox bacteria. Appl. Environ. Microbiol., 2012, 78(3): 752-758. doi.org/10.1128/aem.07113-11.
15. Rotthauwe J H, Witzel K P, Liesack W. The ammonia monooxygenase structural gene amoA as a functional marker: molecular fine-scale analysis of natural ammonia-oxidizing populations. Appl. Environ. Microbiol., 1997, 63(12): 4704-4712. doi.org/10.1126/science.284.5411.63.
16. Throback I N, Enwall K, Jarvis A, Hallin S. Reassessing PCR primers targeting *nirS*, *nirK* and *nosZ* genes for community surveys of denitrifying bacteria with DGGE. FEMS Microbiol. Ecol., 2004, 49(3): 401-417. doi.org/10.1016/s0168-6496(04)00147-3.
17. Enwall K, Philippot L, Hallin S. Activity and composition of the denitrifying bacterial community respond differently to long-term fertilization. Appl. Environ. Microbiol., 2005, 71(12): 8335-8343. doi.org/10.1128/aem.71.12.8335-8343.2005.
18. López-Gutiérrez J C, Henry S, Hallet S, Martin-Laurent F, Catroux G, Philippot L. Quantification of a novel group of nitrate-reducing bacteria in the environment by real-time PCR. J. microbiol. meth., 2004, 57(3): 399–407. doi.org/10.1016/j.mimet.2004.02.009.
